# Mechanism and Regulation of Nucleocytoplasmic Trafficking of Smad

**DOI:** 10.1186/2045-3701-1-40

**Published:** 2011-12-28

**Authors:** Xiaochu Chen, Lan Xu

**Affiliations:** 1 Program in Molecular Medicine, University of Massachusetts Medical School, Worcester, MA USA

## Abstract

Smad proteins are the intracellular mediators of transforming growth factor β (TGF-β) signaling. Smads function as transcription factors and their activities require carboxyl-terminal phosphorylation by TGF-β receptor kinases which are embedded in the cell membrane. Therefore, the translocation of activated Smads from the cytoplasm into the nucleus is a rate-limiting step in TGF-β signal transduction into the nucleus. On the other hand, the export of Smads out of the nucleus turns off TGF-β effect. Such spatial control of Smad ensures a tight regulation of TGF-β target genes. Several cross-talk pathways have been shown to affect TGF-β signaling by impairing nuclear translocation of Smad, exemplifying the biological importance of the nuclear transport process. Many laboratories have investigated the underlying molecular mechanism of Smad nucleocytoplasmic translocation, combining genetics, biochemistry and sophisticated live cell imaging approaches. The last few years have witnessed the elucidation of several key players in Smad nuclear transport, most importantly the karyopherins that carry Smads across the nuclear envelope and nuclear pore proteins that facilitate the trans-nuclear envelope movement. The foundation is now set to further elucidate how the nuclear transport process is regulated and exploit such knowledge to manipulate TGF-β signaling. In this review we will discuss the current understanding of the molecular machinery responsible for nuclear import and export of Smads.

## Introduction

Cytokines of the TGF-β superfamily are critically involved in embryonic development and adult tissue homeostasis [1]. Aberrant TGF-β signaling is a major contributing factor in the pathogenesis of diseases including cancer and tissue fibrosis [2]. Therefore, there has been tremendous interest in understanding how these extracellular factors control gene expression and cellular properties. Genetics identified cell surface TGF-β receptor kinases and intracellular Smad proteins as the critical components of the signaling mechanism [3]. The landmark finding came in the mid 1990s that revealed Smads as transcription factors and that their activities are turned on by direct phosphorylation by the TGF-β receptor kinases [4-7]. These Smads include Smad1, 2, 3, 5 and 8, which are commonly referred to as R-Smad (i.e. receptor-activated Smad). It was immediately realized that R-Smad activation upon TGF-β stimulation is accompanied by their cytoplasm-to-nucleus translocation [5,6]. In this way, the access of Smads to their large number of target genes is strictly signal-dependent, making Smad nuclear translocation an essential step in TGF-β signal transduction into the nucleus.

### The general nuclear transport apparatus

The nuclear envelope restricts the movement of macromolecules between cytoplasm and nucleus, and the exchange of proteins or RNAs between the two compartments is exclusively through the nuclear pore complex (NPC) [8,9]. The prevailing model posits that for proteins to enter or exit the nucleus, they need carrier proteins called karyopherins [9,10]. The association of nuclear import cargoes with their karyopherins (i.e. importins) is disrupted by the small GTPase Ran in its GTP bound form [11]. On the other hand, for karyopherins specialized in nuclear export (i.e. exportins), its binding to cargoes is enhanced by RanGTP [12]. Interestingly Ran GTP is exclusively localized in the nucleus since the Ran GTP exchange factor (i.e. RCC1) is present only in the nucleus. This asymmetric distribution of RanGTP vs. RanGDP across the nuclear envelope ensures directionality in nuclear transport, so that import substrates will unload only in the nucleus while export cargoes accumulate only in the cytoplasm [13]. It was therefore postulated that all karyopherins must have RanGTP binding capability, and this assertion has been validated in almost all cases so far. Over 20 karyopherins have been identified in vertebrates based on the above characteristics [10]. These karyopherin differ significantly in primary amino acid sequence but at least for those whose X-ray crystal structures have been solved the higher order structures are quite similar with multiple HEAT repeats [10]. It is generally presumed that each karyopherin recognizes its specific cargoes for nuclear import or export. For example the lys-rich classic nuclear localization sequence (cNLS), which is present in many nuclear proteins, is directly bound by Imp α and associated with the karyopherin Impβ [14]. The exportin CRM-1 recognizes a well-defined nuclear export sequence (i.e. NES), which is also found in many exported proteins [15]. But for many other karyopherins the sequence elements they recognize have not been well characterized.

While the roles of karyopherins and RanGTP in nuclear transport are well established, how the transport cargoes transit through the NPC remains a highly debatable issue [8,9,16]. The model of NPC function has to simultaneously explain how it serves as a barrier and in the meantime selectively allows trafficking of cargoes. The NPC consists of over 30 evolutionarily conserved nucleoporins, each with particular localization within the NPC [17-19]. Many nucleoporins contain repeats of phenylalanine-glycine (FG), and they align the central tunnel of the NPC. As a result the locally concentrated FG repeats creates a highly hydrophobic environment that restricts movement of macromolecules through the NPC [20]. On the other hand, many of the non-FG nucleoporins assemble into subcomplexes, and they are believed to serve mostly as scaffolds for NPC assembly or anchoring to the nuclear envelope [21-25]. Karyopherins directly interact with FG-nucleoporins, and somehow this overcomes the gating mechanism and allows translocation of cargoes across the NPC. However, exactly how karyopherins and the NPC function in a concerted manner in nuclear transport is still a challenging question that awaits further elucidation [8,9,16,26,27]. Many of these principles in nuclear transport are based on studies of constitutive nuclear import or export events, and it was a question how much of these apply to signal-induced nuclear transport of Smad.

### Nuclear import of Smad

*In vitro *nuclear transport assays and live cell imaging analyses clearly showed that in unstimulated cells Smads (including Smad2, 3 and 4) shuttle in and out of the nucleus constantly [28-31]. These are consistent with the observation that Smads can be found in the nucleus of many cell types in the absence of TGF-β/BMP signaling. Conceivably, the ability of Smads to bind DNA or transcription factors could contribute to their nuclear localization in untreated cells. But the important question is whether such presence of Smads in the nucleus has any significant functional consequences, without C-terminal phosphorylation of R-Smads and complex formation with Smad4. Upon TGF-β/BMP treatment, the C-terminally phosphorylated (i.e. activated) R-Smad becomes exclusively present in the nucleus. Since activated R-Smad is mostly associated with Smad4 in a complex while at basal state R-Smad exists as a monomer, the two forms of Smads may have distinct requirement in order to enter the nucleus.

#### Karyopherin for Smad nuclear import

The current model of nuclear transport was established mostly by biochemical approaches using *in vitro *reconstituted nuclear import assay. While this methodology has revealed many characteristics of Smad nuclear transport as a monomer [32], the difficulty in obtaining pure C-terminal phosphorylated Smad poses a major obstacle to study signal-induced nuclear import of activated Smad as a complex. The technology breakthrough of RNA interference (RNAi) and with it the ability to functionally screen the whole genome, made it feasible to use functional genomics to dissect components of the Smad nuclear import pathway. In an unbiased whole genome screening, the molecule *moleskin *(Msk) was identified as a nuclear import factor of the *Drosophila *Smad homolog MAD [33]. Msk attracted immediate attention because its mammalian homologs were initially identified as Ran-binding proteins and named RanBP7 and RanBP8 [13]. Since Ran-binding is a perceived characteristic of all karyopherins, RanBP7/8 are referred to as Imp7/Imp8 for the belief that they will be found to be karyopherins for certain cargoes. RNAi depletion of Imp7 and Imp8 in mammalian cells strongly inhibited TGF-β or BMP-induced Smad nuclear translocation. Moreover, biochemical studies confirmed that the binding of Smad3 to Imp8 is regulated by RanGTP, suggesting that Smads are *bona fide *cargos of Msk/Imp7/Imp8 [33]. Genetics analysis in the *Drosophila *developing eye further indicated that Msk is the nuclear import factor for Smad *in vivo *[33].

Complementing RNAi-based analysis of Msk and Imp7/8, overexpression experiments showed that an increased level of Imp8 could force nuclear accumulation of Smad without the need for TGF-β stimulation [34]. Interestingly, this only applies to Smad4 and all other R-Smads except for Smad2. This might be due to unique sequence elements in the N-terminal MH1 domain of Smad2 that are absent in other Smads. However, consistent with the fact that R-Smads assemble into heterotrimeric complex with Smad4, it was clear in TGF-β-activated cells Smad2 is imported by Imp7/8, perhaps in a piggyback manner with Smad4.

An issue of debate is the nuclear localization sequence in Smad. Imp8 binds to the MH1 domain of Smad3, which is disrupted by RanGTP [33]. Mutational analysis showed a Lys-rich KKLKK sequence in the MH1 domain that is required for Imp8-driven nuclear import [34]. Surprisingly this KKLKK motif does not appear to be required for Imp8 interaction, prompting the question why this KKLKK motif is so critical for Smad nuclear import [34]. This KKLKK sequence does not function as a classic NLS because it cannot target a heterologous protein into the nucleus, and RNAi experiment conclusively ruled out Imp β (which is responsible for classic NLS-mediated import) as the karyopherin of Smad [34,35]. A recent study identified a phospho-SPS motif in activated ERK that appears to be essential for its binding to Imp7 and nuclear import [36]. A similar but significantly different sequence is also found in the MH2 domain of R-Smad [36]. However it remains to be tested whether phosphorylation of the SPS motif in Smad indeed takes place in TGF-β or BMP treated cells, and whether this is prerequisite and/or sufficient for nuclear import of Smad. Furthermore since the SPS motif in Smad is not located in the region that directly interacts with of Imp7/8, it remains intriguing how this phosphorylation might facilitate Smad nuclear import.

Another unresolved issue concerns the relative contribution of Imp7 and Imp8. While Imp8 alone could drive Smad into the nucleus, Imp7 when expressed at the same level could not accomplish this. Yet, RNAi experiment shows both Imp7 and Imp8 are rate-limiting [34]. Interestingly, while Imp8 could rescue the knockdown of Imp7, Imp7 could not replace the loss of Imp8 [34]. This clearly indicates that these two importins serve non-redundant functions in Smad nuclear transport.

Smad is not likely the only cargo of Msk/Imp7/Imp8. In *Drosophila *and mammalian cells, Msk/Imp7/Imp8 is also important for nuclear import of activated MAPK (mitogen-activated protein kinase) - ERK (extracellular signal regulated kinase), as shown by genetics and biochemistry studies [36,37]. *In vitro *reconstituted import assays also suggest that Imp7/8 transports ribosomal protein and glucocorticoid receptors into the nucleus, but *in vivo *evidence is lacking [38,39]. It is not clear whether these different substrates share a common motif recognized by Imp7/8, or whether the physical interactions are mediated by different domains on Imp7/8. The prospect of ERK and Smad sharing the same karyopherin raises an interesting possibility that the two pathways may compete with each other at the level of nuclear translocation.

#### Nucleoporins involved in Smad nuclear import

Besides Msk/Imp7/Imp8, the most prominent hits in the RNAi screening for Smad nuclear import factors are nucleoporins, including non-FG nucleoporins Sec13, Nup75, Nup93 and Nup205 [35]. A surprise is that knockdown of these nucleoporins apparently had no impact on nuclear import of classic NLS-containing cargoes [35]. Remarkably, fusing a classic NLS to Smad bypassed the need for these non-FG nucleoporins for nuclear import and switched the karyopherin requirement from Msk to Impβ [35]. These observations strongly argue that karyopherins dictate the selection of nucleoporins that mediate the trans-NPC movement of the cargo. Genetics studies in yeast revealed that a subset of FG-nucleoporins are differentially employed by different importins, suggesting specificity among the nucleoporins in terms of which ones are required for different cargoes [40,41]. This also implies that there are multiple routes through which different cargoes migrate through the NPC. So Smad may take a unique route through the NPC.

**Figure 1:**
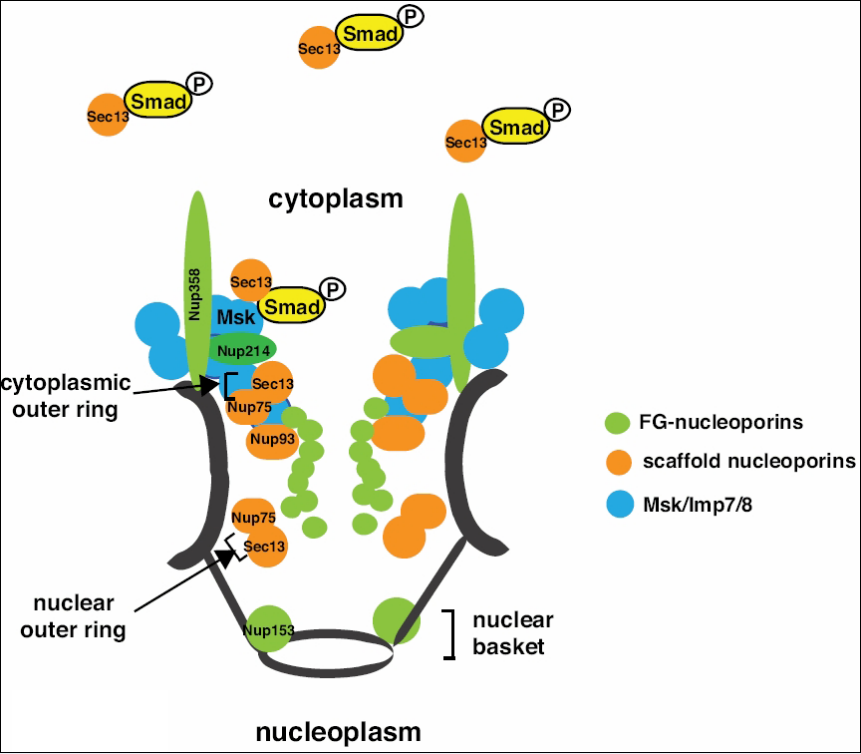
**The molecular machinery for nuclear import of activated Smad**. Shown are Msk/Imp7/8 and the nucleoporins required for nuclear import of Smad, with their relative positions within the NPC indicated. As indicated some of these nucleoporins are located in the cytoplasmic outer ring, the nuclear outer ring, and the nuclear basket sub-structures of the NPC.

Sec13 and Nup93 have properties specific toward Smads. Sec13 interacts much more strongly with C-terminal phosphorylated MAD [35]. This is in contrast to Imp7/8 interaction with Smad which appears to be unaffected by Smad C-terminal phosphorylation [33]. Therefore Sec13 may provide the mechanism through which TGF-β accelerates the nuclear import rate of Smad. It is important to note that, unlike typical nucleoporins which are perceived to be rather stationary and confined within the NPC, Sec13 is dynamic and shuttles between a cytoplasmic pool and the NPC [42]. Nup93 appears to be crucial for Msk localization in cells. In *Drosophila *S2 cells, Msk is concentrated in the nuclear periphery and assumes a "ring" pattern around the nucleus. Depletion of Nup93, but not any of the other nucleoporins that are important for Smad nuclear import, resulted in a much-diffused pattern of Msk distribution [35]. Considering all these features of Sec13 and Nup93, it is tempting to speculate that activated Smad may be associated with Sec13 in the cytoplasm and escorted to the nuclear periphery where it is uploaded onto Msk and transported across the NPC (Figure 1). *In vitro *studies have shown direct physical interaction between Smad and FG- nucleoporins such as Nup153 and Nup214, and indeed their function in Smad nuclear import in intact cells was confirmed by RNAi experiments [28,35]. In addition, Nup358 was found to be required for concentrating Msk to the nuclear periphery, similar to the function of Nup93. Nup358 is located at the cytoplasmic face of the NPC and extends into the cytoplasm. It may therefore act as a capturing device to recruit Msk/Imp7/8 to the vicinity of NPC.

Sec13, Nup75 and Nup93 are believed to act as scaffold in the NPC, thus their seemingly specific requirement in Smad nuclear import is unexpected. Another interesting aspect is that nucleoporins, including Sec13 are recently shown to associate with chromatin and directly play a role in transcriptional regulation [43,44]. The physical interaction between Sec13 and activated Smad thus raises an intriguing possibility that beyond nuclear translocation, Sec13 may also contribute to targeting Smads to their binding sites in the chromatin and participate in Smad-mediated transcriptional regulation.

### Nuclear export of Smad

Nuclear export of Smad attracted a lot of attention as it presents a mechanism that terminates TGF-β signaling in the nucleus. Among all Smads, only Smad4 contains a classic Leu-rich NES that is recognized by the export receptor CRM-1 [45,46]. In unstimulated cells, CRM-1 acts to maintain Smad4 in the cytoplasm and without CRM-1 function Smad4 spontaneously accumulate in the nucleus. It is very clear that R-Smads are not regulated by CRM-1, as their distribution is unchanged upon leptomycin B (a CRM-1 inhibitor) treatment [29]. Smad4 becomes restricted in the nucleus upon TGF-β stimulation, because the NES sequence of Smad4 is masked upon association with C-terminal phosphorylated R-Smad [47]. Therefore, only decommissioned Smad4, i.e. those dissociated from R-Smad, is readily exported out of the nucleus.

After a systematic analysis of all known karyopherins, Kurisaki et al. identified exportin 4 as capable of interacting with Smad3 in a RanGTP regulated manner [48]. In *in vitro *reconstituted assays as well as RNAi experiment testing endogenous proteins, exportin 4 acted as an export factor for Smad3. As expected, the interaction between Smad3 and exportin 4 is diminished upon C-terminal phosphorylation of Smad3, so blocking the export is an important aspect in nuclear accumulation of Smad3 in response to TGF-β. More recently, RanBP3 was proposed as another export factor of Smad2 and Smad3 [49]. Many features of RanBP3 interaction with Smad are similar to those found in exportin 4-Smad interaction, such as that dephosphorylation of Smad is important for the interaction to take place. For both exportin 4 and RanBP3, other export cargoes have also been described, including eIF-5A (for exportin 4) and β-catenin (for RanBP3) [50,51]. Moreover, RanBP3 is also well known as a cofactor of CRM-1 which exports many proteins [52]. Exportin 4 and RanBP3 do not share strong sequence similarity so it is unclear whether they export Smad through different mechanisms and whether they are employed in different cellular contexts. Like nuclear import, the translocation from nucleus to cytoplasm involves coordinated action of the exportins and nucleoporins. Whether nuclear export of Smad entails a unique set of nucleoporin awaits further investigation.

#### Intracellular movement of Smad by motor proteins

Another important aspect of Smad dynamics in the cell is motor proteins that have been implicated in Smad trafficking between the cell membrane and the nucleus. Microtubules and the associated motor proteins such as kinesin have been shown to recruit Smad to the receptor kinase [53]. Dynein light chain protein km23-1 (DYNLRB1) plays a role in Smad movement toward the nucleus after activation by the receptor kinases [54]. These reports support the notion that Smad movement within the cell is an actively facilitated process rather than passive diffusion.

### Regulatory Mechanisms Impinged on Smad Nucleocytoplasmic trafficking

Nuclear translocation and hence the level of Smad signaling into the nucleus is regulated by multiple factors, often with profound biological consequences [55].

#### Smad modification

While the C-terminal phosphorylation is prerequisite for Smads to accumulate in the nucleus, there are phosphorylation events that inhibit Smad nuclear translocation. Phosphorylation in the linker region, mediated by CDKs (cyclin-dependent kinase) and MAPK, reduced Smad nuclear accumulation [56,57]. In *Drosophila*, phosphorylation in the MH1 domain of MAD by NLK (nemo-like kinase) results in diminished nuclear concentration of MAD [58]. With knowledge on the karyopherins and nucleoporins involved in nuclear transport, it should be straightforward to test the underlying mechanism of such inhibitions. For example, since the MH1 domain of Smad interacts with Imp7/8, it would be interesting to test whether NLK phosphorylation would interfere with this interaction and hence impede Smad nuclear import. On the other hand, phosphorylation by NLK may regulate Smad interaction with other factors that also contribute to Smad nuclear accumulation, such as nucleoporins, nuclear retention factors, etc. Phosphorylation of the SPS motif in the MH2 domain of R-Smad was proposed to be important for Smad nuclear accumulation [36], but the question of which kinase and signaling pathway is responsible for this phosphorylation remains unanswered.

Sumoylation and ubiquitination of Smads also affect nuclear accumulation of Smads. For example, in mammalian cells, the mono-ubiquitination status of Smad4 is controlled by the mono-ubiquitin ligase TIF1γ and a deubiquitinase FAM (USP9x) [59]. Mono-ubiquitination of Smad4 inhibits its association with activated Smad2 and reduces Smad4 concentration in the nucleus, presumably because monomeric Smad4 is unmasked and readily exported by CRM-1[59]. In *Drosophila *the Smad4 homolog Medea is sumoylated, which enhances nuclear export of Medea and thereby limits the level of TGF-β signaling in the developing embryo [60]. In this case, however, how sumoylation of Smad4 facilitates its nuclear export is unclear.

#### Regulation of karyopherins

Genetic analysis in *Drosophila *found that the ability of Msk to transport ERK into the nucleus depends on integrin [61]. In integrin mutant expressing cells, while ERK is properly activated and Msk is present, ERK concentrates around the nuclear periphery and unable to translocate through the NPC [61]. This and other evidence suggest that perhaps Msk needs to be activated in order to transport ERK into the nucleus. Interestingly, in *Drosophila *cells Msk is phosphorylated on tyrosine residues, and the level of phosphorylation is decreased in integrin mutant expressing cells [61]. Moreover, a phospho-tyrosine phosphatase Corkscrew binds to Msk and appears to target a subpopulation of Msk to the cell cortex [61]. These observations raise the intriguing possibility that Msk activity may be regulated by integrin signaling, but the underlying molecular mechanism is a completely open question. The intriguing question is whether nuclear import of Smad also requires activation of Msk. It is also possible that different forms of Msk may be engaged in nuclear transport of different cargoes. These are very important questions that await further investigation.

John Blenis' group made an interesting observation that kinases such as RSK and Akt in the Ras and PI3K pathways can directly phosphorylate RanBP3 on Ser58 [62]. More importantly, this phosphorylation increased RanBP3 binding to Ran and resulted in reduced nuclear import of cNLS cargoes. So whether and how Ser58 phosphorylation of RanBP3 would affect Smad nuclear export and/or import deserves further investigation.

#### TAZ and YAP

In the nucleus, Smad is often associated with other DNA-bound transcription factors. Overexpression of some of these Smad cofactors, such as FoxH1 and ATF2, could influence Smad retention in the nucleus [63,64]. However such an effect may be an artifact of overexpression, and whether endogenous FoxH1 and ATF2 do indeed regulate Smad localization is debatable.

Recently, RNAi knockdown of endogenous proteins provided compelling evidence that the transcription factor YAP (Yes associated protein) and TAZ (transcriptional co-activator with PDZ-binding motif) are required for TGF-β-induced nuclear accumulation of Smads without apparently affecting Smad C-terminal phosphorylation [65]. YAP and TAZ are DNA bound transcription factors exclusively present in the nucleus. YAP and TAZ are phosphorylated in response to activation of the Hippo pathway and as a consequence driven out of the nucleus [66,67]. Indeed when YAP and TAZ become localized to the cytoplasm in Hippo-activated cells, TGF-β failed to induce nuclear accumulation of Smad even though Smad C-terminal phosphorylation appeared to be intact [68]. Why the presence of YAP and TAZ in the nucleus is so important for Smad nuclear accumulation is not immediately clear. YAP and TAZ directly interact with Smad, so one possibility is that when YAP and TAZ are in the cytoplasm they sequester Smad in a way that prevents Smad association with Imp7/8 or nucleoporins for nuclear import. But this idea does not explain why depletion of TAZ by RNAi also prevents Smad translocation into the nucleus [65]. Maybe the association of YAP and Smad in the nucleus prevents interaction with exportin 4 or RanBP3. The Hippo pathway regulates organ size in response to cell density, so it is interesting that this pathway has a critical impact on TGF-β/BMP signaling into the nucleus. With important biological implications in the cross-talk between the Hippo and TGF-β/BMP pathways, there is considerable interest in understanding how YAP and TAZ control Smad nuclear accumulation.

## Conclusion

Signal-induced nuclear translocation of key molecules is a common phenomenon in many signaling pathways, and what is learned about Smad nucleocytoplasmic trafficking will shed light on this important cell biology process. We now know much about the molecular mechanism of nucleocytoplasmic transport of Smad. But how such regulation may impact the biological functions of TGF-β/Smad remains to be investigated. In embryonic development, TGF-β/BMP act as morphogens and the level of signals transduced into the nucleus is translated into distinct cell fate determination. It is conceivable that the Smad nuclear import or export machinery may play a role in such regulation. Furthermore whether the transport machinery may be dysregulated in various disease pathogenesis also remains to be explored. Finally, given the knowledge on Smad nuclear import and export machinery, we are in a good position to design novel approaches that allow manipulation of Smad signaling into the nucleus.

## Competing interests

The authors declare that they have no competing interests.

## Authors' contributions

XC and LX jointly wrote the manuscript, both have read and approved the final manuscript.
